# Exposure risk analysis of COVID-19 for a ride-sharing motorbike taxi

**DOI:** 10.1063/5.0069454

**Published:** 2021-11-17

**Authors:** R. Hetherington, A. B. M. Toufique Hasan, A. Khan, D. Roy, M. Salehin, Z. Wadud

**Affiliations:** 1 School of Civil Engineering, University of Leeds, Leeds LS2 9JT, United Kingdom; 2 Department of Mechanical Engineering, Bangladesh University of Engineering and Technology (BUET), Dhaka, Bangladesh; 3 Institute for Transport Studies and School of Chemical and Process Engineering, University of Leeds, Leeds LS2 9JT, United Kingdom

## Abstract

A dominant mode of transmission for the respiratory disease COVID-19 is via airborne virus-carrying aerosols. As national lockdowns are lifted and people begin to travel once again, an assessment of the risk associated with different forms of public transportation is required. This paper assesses the risk of transmission in the context of a ride-sharing motorbike taxi—a popular choice of paratransit in South and South-East Asia and Sub-Saharan Africa. Fluid dynamics plays a significant role in understanding the fate of droplets ejected from a susceptible individual during a respiratory event, such as coughing. Numerical simulations are employed here using an Eulerian–Lagrangian approach for particles and the Reynolds-averaged Navier–Stokes method for the background air flow. The driver is assumed to be exhaling virus laden droplets, which are transported toward the passenger by the background flow. A single cough is simulated for particle sizes 1, 10, 50 μm, with motorbike speeds 1, 5, 15 m/s. It has been shown that small and large particles pose different types of risk. Depending on the motorbike speed, large particles may deposit onto the passenger, while smaller particles travel between the riders and may be inhaled by the passenger. To reduce risk of transmission to the passenger, a shield is placed between the riders. The shield not only acts as a barrier to block particles, but also alters the flow field around the riders, pushing particles away from the passenger. The findings of this paper therefore support the addition of a shield potentially making the journey safer.

## I. INTRODUCTION

In March of 2020, the World Health Organization (WHO) declared the spread of SARS-CoV-2 to be a global pandemic. National lockdowns have been implemented globally to reduce the spread of the COVID-19 virus. However, once a lockdown is lifted and people return to work and social events, an understanding of transmission within public transport is required to allow for a safe return.

Virus laden fluid droplets are generated in the respiratory tract during respiratory events, such as breathing, talking, coughing, and sneezing. The mechanisms of droplet formation will not be reviewed here; instead the reader is directed to^1^ Sec. II A, and references therein. Once expelled from an infected host, droplets typically take three routes to a susceptible individual:^1^ (i) contact with surfaces onto which droplets have been deposited, (ii) ballistic projection of large droplets directly onto a susceptible individual, and (iii) inhalation of small virus laden droplets, otherwise referred to as aerosols. Here, the definition of an aerosol follows that of Fennelly:^2^ “a suspension of fine solid particles or liquid droplets in air with a small diameter typically less than 5 *μ*m.”

In the early stages of the pandemic, airborne transmission—defined as the spread of an infectious agent caused by the dissemination of aerosols that remain infectious when suspended in air over long distances and time^3^—was not believed to make a significant contribution to the transmission of COVID-19. A scientific brief from the WHO in March 2020 did not recognize the role of transmission through aerosols.^4^ However, a group of 239 scientists signed a letter to the WHO to highlight the importance of considering the airborne transmission route of aerosols,^5^ and the WHO subsequently updated their advice in July 2020.^6^ Airborne transmission has since been confirmed as the dominant mode of transmission,^7,8^ and retrospective analysis of “spreader-events” has confirmed the prevalence of airborne transmission. For example, an outbreak of COVID-19 between three separate tables in a restaurant in Guangzhou China occurred in January 2020.^9^

Ventilation is key to provide fresh air and dilute the concentration of an airborne virus. In indoor public spaces, it is widely accepted that a 2 m (or 6 ft) social distancing measure is sufficient because large droplets will fall to the floor before reaching the susceptible individual, and aerosols will be transported away via diffusion. The 2 m rule assumes a quiescent background flow; however, this is an exception rather than a rule and depends on many human and environmental factors. Even a simple action such as walking produces a complex recirculating flow behind the infected individual.^10^ An accurate prediction of the background flow is essential before considering droplet transport; therefore, a 2 m rule cannot be universal and must be adjusted based on the particular flow conditions.

Computational fluid dynamics (CFD) can play a significant role in our understanding of airborne transmission due to environmental variables, such as background air currents and turbulence.^11–15^ A number of recent CFD studies have employed the Eulerian–Lagrangian approach to modeling airflow and particle dispersion.^13,16–19^ Here, the term “particle” is used to describe droplets of all sizes, including aerosols. The governing equations of fluid flow are first solved in the Eulerian frame of reference to obtain the velocity field of the continuous phase, most commonly via the Reynolds-averaged Navier–Stokes (RANS) equations. Particles are subsequently tracked through the domain in a Lagrangian framework by considering a force balance on the particle.

CFD has also been applied to other methods of transportation, such as university campus buses^20^ and the London underground.^21^ In many developing and emerging South and South-East Asian and Sub-Saharan African countries, such as Bangladesh, Vietnam, Indonesia, Uganda, Nigeria, Rwanda, etc.,^22^ ride-sharing motorbike taxis are a popular mode of public transport. In order to combat the spread of COVID-19, motorcycle taxis have been closed in most of these countries. This has caused a detrimental effect on the livelihoods of a large number of economically disadvantaged people—both the drivers and the passengers—causing immense economic hardship and social consequences. However, to the authors' best knowledge, an assessment of airborne transmission has not been carried out in the literature for the motorbike taxi geometry. This is an important gap in the literature to address—a very large number of people rely on motorbike taxis in the aforementioned region, and there is a need for understanding their risks and potentially introducing safety measures to reduce those risks.

This paper investigates risks associated with airborne transmission for the case of a susceptible passenger riding with an infected driver. Airflow and particle modeling is carried out using the Eulerian–Lagrangian approach. A cough is simulated from the driver's mouth with particles of diameters 1, 10, and 50 μm.^13,17,23,24^ This covers a wide range of particle behavior, from ballistic projection to fine aerosol transportation.^1^ Typical motorbike taxi speeds around a busy city depend on the prevailing traffic conditions and are therefore variable, so here 1, 5, and 15 m/s (3–55 km/h) will be tested as a reasonable range. A shield will be considered as a barrier between driver and passenger as a possible method to reduce exposure to the virus. Two factors of transmission are explored: deposition on surfaces, and proximity to the passenger's mouth (i.e., via inhalation).

## II. NUMERICAL PROCEDURE

### A. Problem description

**FIG. 1.**
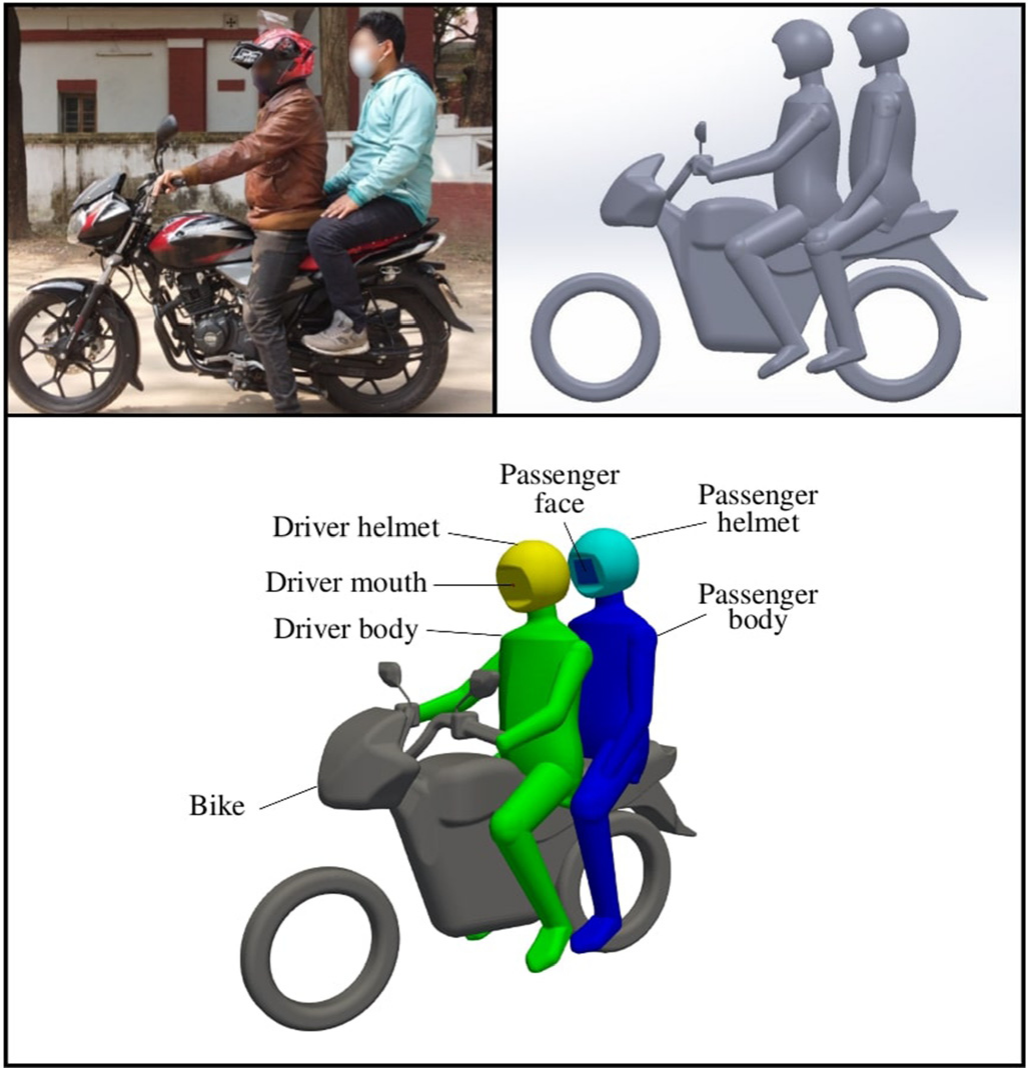
Top left: Bajaj Discover 125 cc bike. Top right: CAD geometry. Bottom: patches defined to assess deposition.

A typical motorbike taxi is considered in this study with one driver and one passenger. The motorbike is based on the Bajaj Discover 125cc bike, which is a popular motorbike in Bangladesh and Sub-Saharan African countries. A computer-aided design (CAD) is constructed using images of a driver and passenger sat on the bike in a static position (see Fig. 1). Simplifications to the CAD have been made in order to reduce the number of cells required to mesh the geometry. Small features, such as disk brake calipers, suspension forks, and wheel spokes, have been omitted because they are expected to make little difference to the flow field around the riders, but will significantly increase the computational cost. Similar geometric simplifications were adopted in several recent aerodynamic studies.^25,26^ Surfaces on the driver and passenger are grouped into patches to provide detailed information on where deposition occurs in the solution log files.

**FIG. 2.**
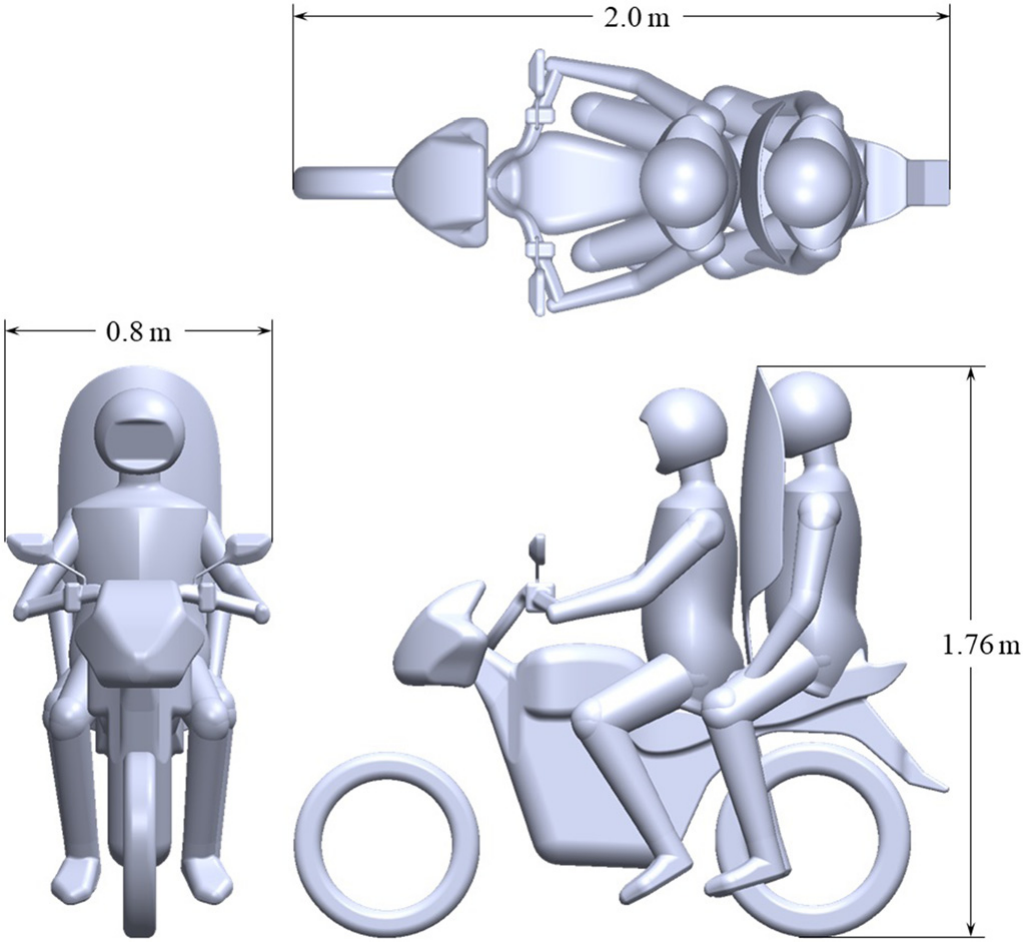
Top, front, and side view of the CAD geometry, including shield.

A curved shield is constructed between the driver and passenger, as shown in Fig. 2. The primary goal of the shield is to block particles, therefore reducing deposition onto the passenger. However, it is unclear at this stage how the shield will alter the flow field around the riders. Further questions on shield design, which will not be considered in this study, are (i) material, (ii) fixing point to the bike/rider, (iii) rigidity, and (iv) stability.

### B. Continuous phase

The open source finite volume method (FVM) code of OpenFOAM^27^ is used to discretise and solve the governing equations of incompressible fluid flow. Particles are transported by a carrier phase (air), which is simulated in the present study using the RANS equations of mass and momentum conservation, given by

$$1\nabla \cdot \bar{u}=0,$$ (1)

$$\frac{\partial \bar{u}}{\partial t}+(\bar{u}\cdot \nabla )\bar{u}=-\nabla \bar{P}+\nu \nabla^{2}\bar{u}-\nabla (\bar{u\prime u\prime }),$$ (2)

where $\bar{u}$ and $u^{\prime }$ are the Reynolds-averaged and fluctuating velocity, respectively, $\bar{P}$ is the kinematic pressure, and *ν* is the kinematic viscosity. A Cartesian coordinate system is adopted, with streamwise coordinate *x*, normal direction *y*, and vertical component *z*. Closure for (2) is achieved through modeling the Reynolds stress term $\left(\bar{u\prime u\prime }\right)$ by a turbulence model and most commonly by the addition of the turbulent viscosity hypothesis.

The popular *k*-*ω* SST model of Menter^28–30^ is chosen for the current study as a suitable model for the prediction of aerodynamic flows with strong adverse pressure gradients and separation. The amended coefficient model^31^ is used in this investigation and provided as a stock model in OpenFOAM. Scales of turbulence are calculated from transport equations for turbulent kinetic energy *k* and specific dissipation *ω*. Further details of the model are omitted here because it has been described in numerous books and technical papers.^32,33^ Additionally, implementation in OpenFOAM can be accessed through the user guide.^34^

### C. Discrete phase

Particles are assumed to be inert; therefore, no heat transfer or evaporation models are required. Instead of allowing for evaporation, a range of diameters are investigated separately. Three diameters are selected: $D_{p}=1,10,50\;\mu m$. These sizes have been chosen to capture the different behavior produced from aerosols which follow the flow and can remain suspended for a matter of hours to large particles which behave like ballistic projectiles.^13,17,23,24^

Particles are injected into the domain via the driver's mouth using the patch injection function of OpenFOAM. A cough is modeled based on the parameters used in Dbouk and Drikakis,^13^ Hossain and Faisal,^17^ and Xie *et al.*^35^ A mouth is defined by an opening of $1.5\times 10^{-4}\;m^{2}$ in the CAD model, in close agreement with Dbouk and Drikakis^13^ who used high-speed photography to find a maximum mouth opening during coughing of $1.9\times 10^{-4}\;m^{2}$. Table I presents cough and particle properties used in this study.

**TABLE I.** Properties used to simulate a single cough. Particle density is approximated by the density of water at 20 °C.

Particles	Diameter $D_{p}$	1,10,50 μm
	Density $\rho_{p}$	$1000\;\text{kg}/m^{3}$
Cough	Duration	0.3 s
	Number of particles $N_{p}$	1004
	Velocity	10 m/s

Surfaces extracted from the CAD model (see Fig. 1) are defined as “stick” surfaces in the simulation setup, such that once particles come into contact with a surface, they do not rebound and re-suspend. A log file is produced from the CFD simulations for each time step which details the number of particles which have deposited onto each surface.

The volume fraction of particles is estimated by considering the volume of fluid ejected from the mouth during a cough, denoted $V_{e}$. For an ejection speed of 10 m/s over a duration 0.3 s, a mouth opening $1.5\times 10^{-4}\;m^{2}$ generates a volume of $V_{e}=4.5\times 10^{-4}\;m^{3}$. A total of $N_{p}=1004$ particles are injected into the domain. Each particle has a volume $V_{p}$, given by the volume of a sphere; therefore, the volume fraction is given by $\alpha =N_{p}V_{p}/V_{e}$. For the largest particles investigated here ( $D_{p}=50\;\mu m$), this yields a volume fraction of $1.5\times 10^{-7}$. For the particles considered in this study, the volume fraction is therefore considerably low ( $<10^{-6}$), such that there is no coupling from particles back to fluid.^36^ A one-way coupled Eulerian–Lagrangian approach is therefore suitable for the current application. It is noted here that particles of diameter $D_{p}=100\;\mu m$ yield a volume fraction of $\alpha =1.2\times 10^{-6}$, which according to the classification map of Elghobashi^36^ require a two-way coupling between particles and fluid.

Using a Lagrangian approach, a set of differential equations are constructed from a force balance to compute the particle position and velocity, according to

$$1\frac{dx_{p}}{dt}=u_{p},$$ (3)

$$m_{p}\frac{du_{p}}{dt}=\sum F_{i},$$ (4)

where $x_{p}$ is the position vector of the particle, $u_{p}$ is the particle velocity, $m_{p}$ is the particle mass, and $F_{i}$ contains body forces on the particle such that

$$\sum F_{i}=F_{D}+F_{B}+F_{G},$$ (5)

where $F_{D},\;F_{B}$, and $F_{G}$ are drag, buoyancy, and gravitational forces, respectively. Buoyancy and gravitational forces are given by

$$F_{B}+F_{G}=\frac{(\rho_{p}-\rho )\pi D_{p}^{3}}{6}\;g,$$ (6)

where $\rho_{p}$ is the particle density, *ρ* is the fluid density, and $g\equiv (0,0,-9.81)\;m/s^{2}$ is the gravitational vector. In OpenFOAM, drag on a particle is approximated by

$$F_{D}=\frac{3}{4}\frac{\rho }{\rho_{p}}\frac{m_{p}}{D_{p}}C_{D}(u-u_{p})|u-u_{p}|,$$ (7)

where $u=\bar{u}+u\prime$ is the instantaneous velocity, and *C_D_* is the drag coefficient for a sphere, given by

$$C_{D}=\{\begin{array}{cc} \frac{24}{Re_{p}}\left(1+\frac{1}{6}Re_{p}^{2/3}\right), & Re_{p}\leq 1000;\\ 0.424, & Re_{p}>1000, \end{array}$$ (8)

where $Re_{p}=D_{p}(u_{p}-u)/\nu$ is the particle Reynolds number.

An estimation of the fluctuating velocity u′ is required in order to obtain the instantaneous velocity ***u*** from the Reynolds-averaged velocity $\bar{u}$. In OpenFOAM, the stochastic dispersion model for RANS takes the form

u′=|γ|dσ, (9)

where *γ* is a scalar sampled from a unit normal distribution, ***d*** is a vector with random direction distributed uniformly in spherical coordinates, and *σ* is the variance, calculated from the kinetic turbulent energy: $\sigma =\sqrt{2k/3}$.

### D. Solution control

**FIG. 3.**
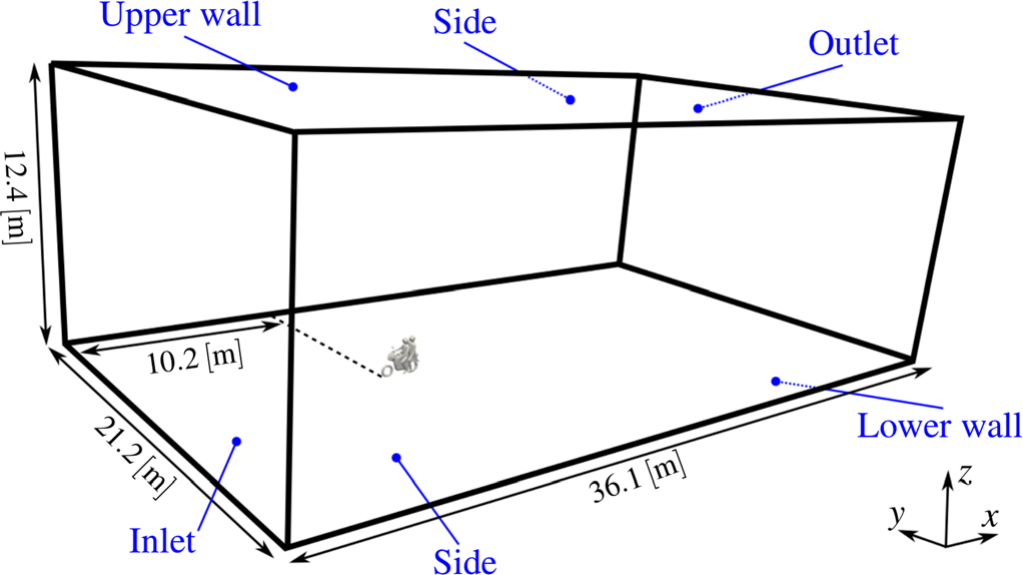
Computational domain and boundary patch names.

**FIG. 4.**
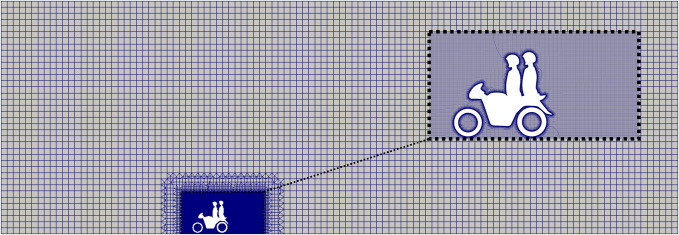
Slice through the computational mesh along the mid-plane (*y *=* *0) for the case of the motorbike taxi without a shield.

Figure 3 presents the computational domain and coordinate system. The inlet is set 10.2 m upstream from the bike's front wheel, and an outlet 25.9 m from the back wheel. The domain width and height are 21.2 and 12.4 m, respectively. This domain size has been taken from the combined numerical and experimental study of Blocken *et al.*,^37^ which is used to validate the current meshing and solution control. See Appendix for further details (Fig. 4).

A uniform flow is prescribed at the inlet along the streamwise direction. Pressure is specified at the inlet using a zero gradient Neumann boundary condition, and at the outlet using a Dirichlet fixed value of $P=0\;m^{2}/s^{2}$. Negligible turbulence intensity ( $k=1\times 10^{-6}\;m^{2}/s^{2}$) is set at the inlet; therefore, the inflow is assumed to be laminar. The top, bottom, and side walls are all treated as slip boundaries. Table II presents a summary of the boundary conditions. The “gap approach” is followed here, where the motorbike geometry is translated 50 mm in the vertical direction, such that there is no contact between wheel and ground. This approach has been used previously by researchers and found to have little impact on results.^38^

**TABLE II.** Boundary patch prescriptions. Freestream values: $U_{\infty }=1,5,15\;m/s,\;p_{\infty }=0\;m^{2}/s^{2},\;k_{\infty }=1\times 10^{-6}\;m^{2}/s^{2}$.

Patch	*u* (m/s)	*P* $\left(m^{2}/s^{2}\right)$	*k*
Inlet	$\left(U_{\infty },0,0\right)$	∇P=0	$k_{\infty }$
Outlet	inletOutlet	0	inletOutlet
Bike/riders	No slip	∇P=0	0
Lower wall	Slip	Slip	Slip
Upper wall	Slip	Slip	Slip
Side patches	Slip	Slip	Slip

A computational mesh is generated in the OpenFOAM application of snappyHexMesh. snappyHexMesh takes a coarse orthogonal background mesh, snaps to the surfaces of the geometry, refines cells near the surface, and builds layers from the geometry walls. Following Blocken *et al.*,^37^ the first layer thickness is set to 60 μm around the bike, riders, and shield. An average value of $y^{+}<1$ is reported on all surfaces. For the case with no shield, a mesh size of 40.2 × 10^6^ cells is generated. With the shield, a mesh size of 44.9 × 10^6^ is required.

Once a mesh has been generated, the first stage to running simulations is to compute the background fluid flow. To initialize each flow field, a potential flow solution is generated using the potentialFoam solver. Pressure and momentum are coupled by the SIMPLEC algorithm of Van Doormaal and Raithby.^39^ Each simulation is ran for a total of 5000 iterations. A pseudo time step of $t=1\times 10^{-6}\;s$ is enforced at each iteration. Second-order discretization schemes are used for convective and viscous terms. Linear interpolation is used to project variables from cell centers to cell faces. A normalized residual error of $1\times 10^{-8}$ has been enforced for each variable.

The second stage of each simulation is to inject particles into the domain, which are transported by the background fluid flow. The OpenFOAM Lagrangian solver DPMFoam solves for a kinematic particle cloud. DPMFoam has been validated against experimental data sets of particle dispersion in separated turbulent flow.^40^ A time step of $t=1\times 10^{-4}\;s$ is enforced to evolve particles in time. There is no coupling from the discrete phase to the continuous phase. Simulations are undertaken on ARC4, part of the High Performance Computing (HPC) facilities at the University of Leeds, UK. The domain is decomposed into 40 sub-domains and ran in parallel.

## III. RESULTS AND DISCUSSION

### A. Aerodynamic properties

**FIG. 5.**
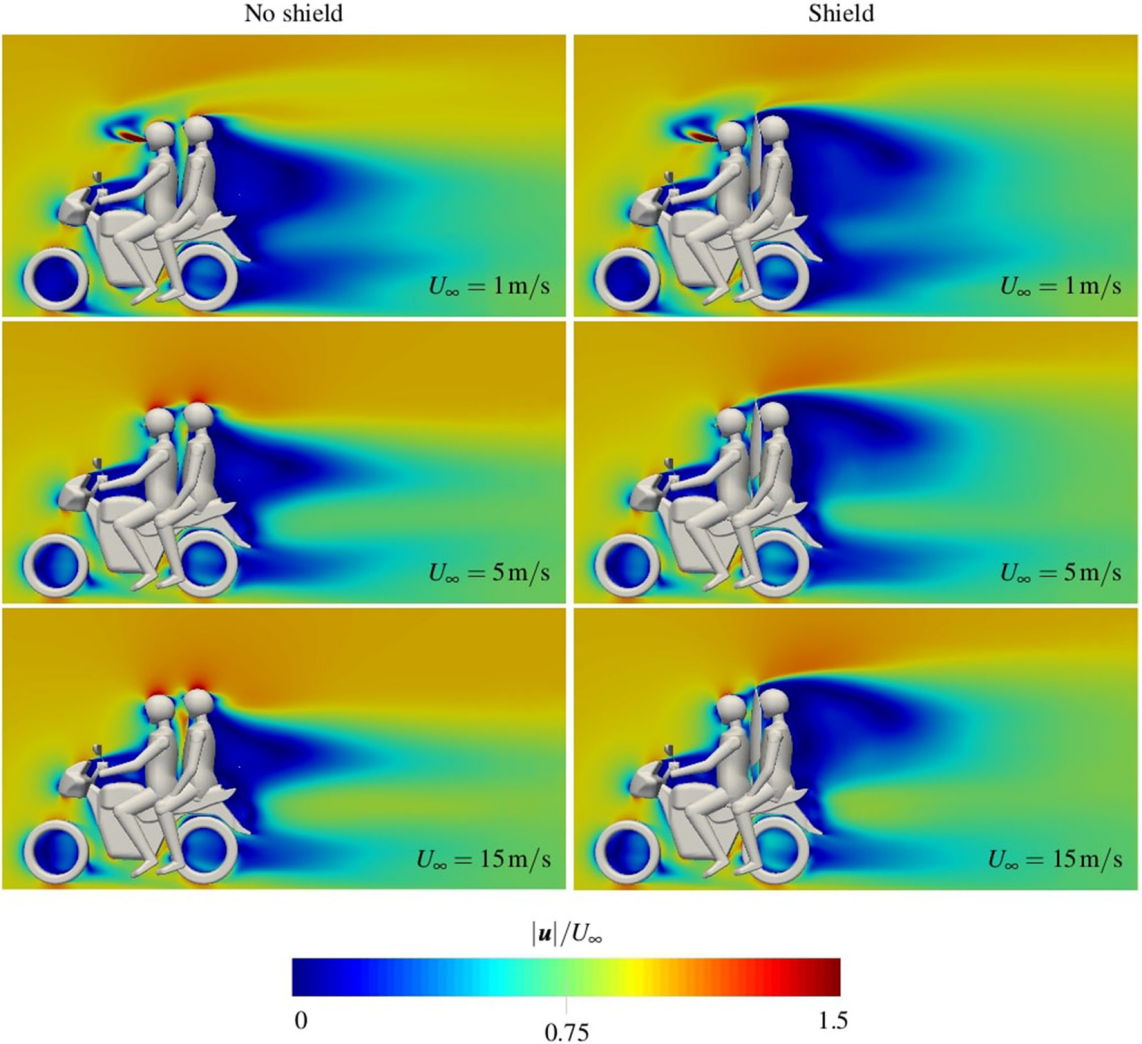
Contours of normalized mean velocity $|U|/U_{\infty }$ along the mid-plane *y *=* *0.

Contours of normalized mean velocity are presented in Fig. 5 along the mid-plane. Simulations show large flow separation behind the passenger and bike. With the addition of a shield, the separation region increases in size, especially in the region behind the passenger's helmet. At the lowest inlet speed of 1 m/s, the cough is more pronounced, as evident in the prominent jet seen in Fig. 5. As the inlet speed is increased, the jet becomes dominated by the bulk streamwise flow. There is no visible jet for an inlet speed of 15 m/s.

**FIG. 6.**
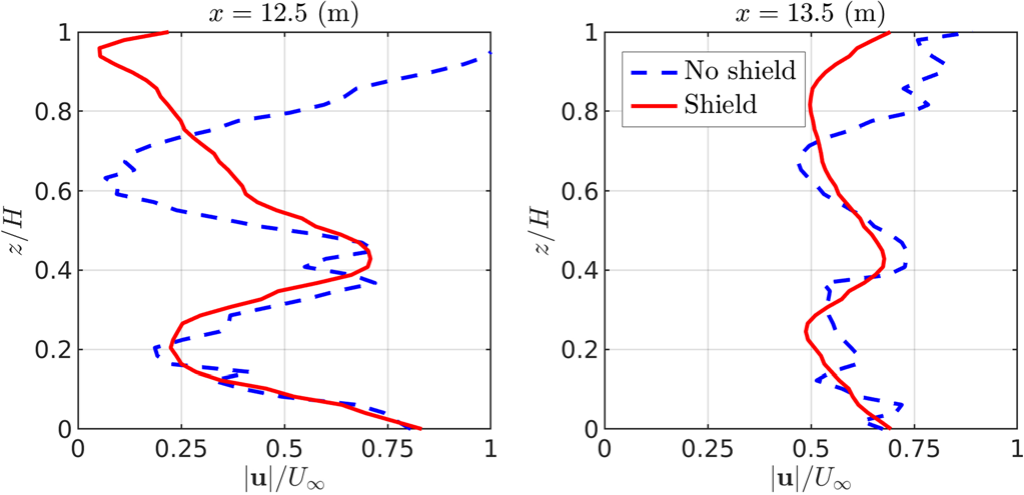
Velocity in the motorbike wake for an inlet speed of 5 m/s. Profiles recorded along two vertical lines spanning 0≤z/H≤1 at downstream position x=12.5 and x=13.5 m. Here, H=1.76 m is the geometry height, as displayed in Fig. 2.

In Fig. 6, velocity magnitude is plotted in the motorbike's wake as a function of vertical coordinate *z*. Height is non-dimensionalised by the geometry height H=1.76 m (see Fig. 2). An inlet speed of 5 m/s is considered for motorbike geometries with and without a shield. Two downstream positions are chosen close to the bike: x=12.5,13.5 m. These locations are in close proximity to the motorbike geometry, which extends from x=10.2 to x=12.2 m in the streamwise direction. Close to the ground ( z/H=0), profiles of velocity with and without the shield are similar. The shield does not significantly alter the wake at this height. However, in the near wake at x=12.5 m, significant deviation is present in the velocity profiles at heights of z/H>0.4. For the case of no shield, velocity has almost recovered to the freestream value at z/H=1. With a shield in place, velocity approaches zero. The shield can therefore be seen to significantly alter wake dynamics near the riders.

The flow field between the riders is important to characterize because it determines the fate of particles which fall between the rider and passenger. Large velocities are reported between the rider and passenger when there is no shield present. Large values of velocity magnitude arise from the vertical component $\left|u_{z}\right|$, which is a factor ∼5 times larger than $\left|u_{x}\right|$, and a factor ∼40 times larger than $\left|u_{y}\right|$. Additionally, *u_z_* is predominantly negative; therefore, a considerable downdraught is produced between the riders.

The drag coefficient $C_{D}^{\prime }$ is written out at each time step, from which it is possible to calculate the drag force using

$$F_{D}^{\prime }=\frac{1}{2}\rho A_{\text{ref}}C_{D}^{\prime }U_{\infty }^{2},$$ (10)

where $\rho =1.18\;\text{kg}/m^{3}$ is the density of air at 20 °C, $A_{\text{ref}}$ is the reference area obtained from the CAD software, and $U_{\infty }$ is the bulk flow or inlet speed. For the no shield case, $A_{\text{ref}}=0.75\;m^{2}$, and for the shield case $A_{\text{ref}}=0.83\;m^{2}$. From simulation start-up, it takes approximately 1000 iterations for the time trace of $F_{D}^{\prime }$ to become approximately constant. Profiles display some fluctuation due to unsteadiness in the flow field; therefore, $F_{D}^{\prime }$ is averaged across the window of iteration 1000 to 5000. An increase in drag of 24% is observed when the shield is added from a reported value of 77.7 N with no shield to 96.0 N with a shield.

### B. Deposition

**FIG. 7.**
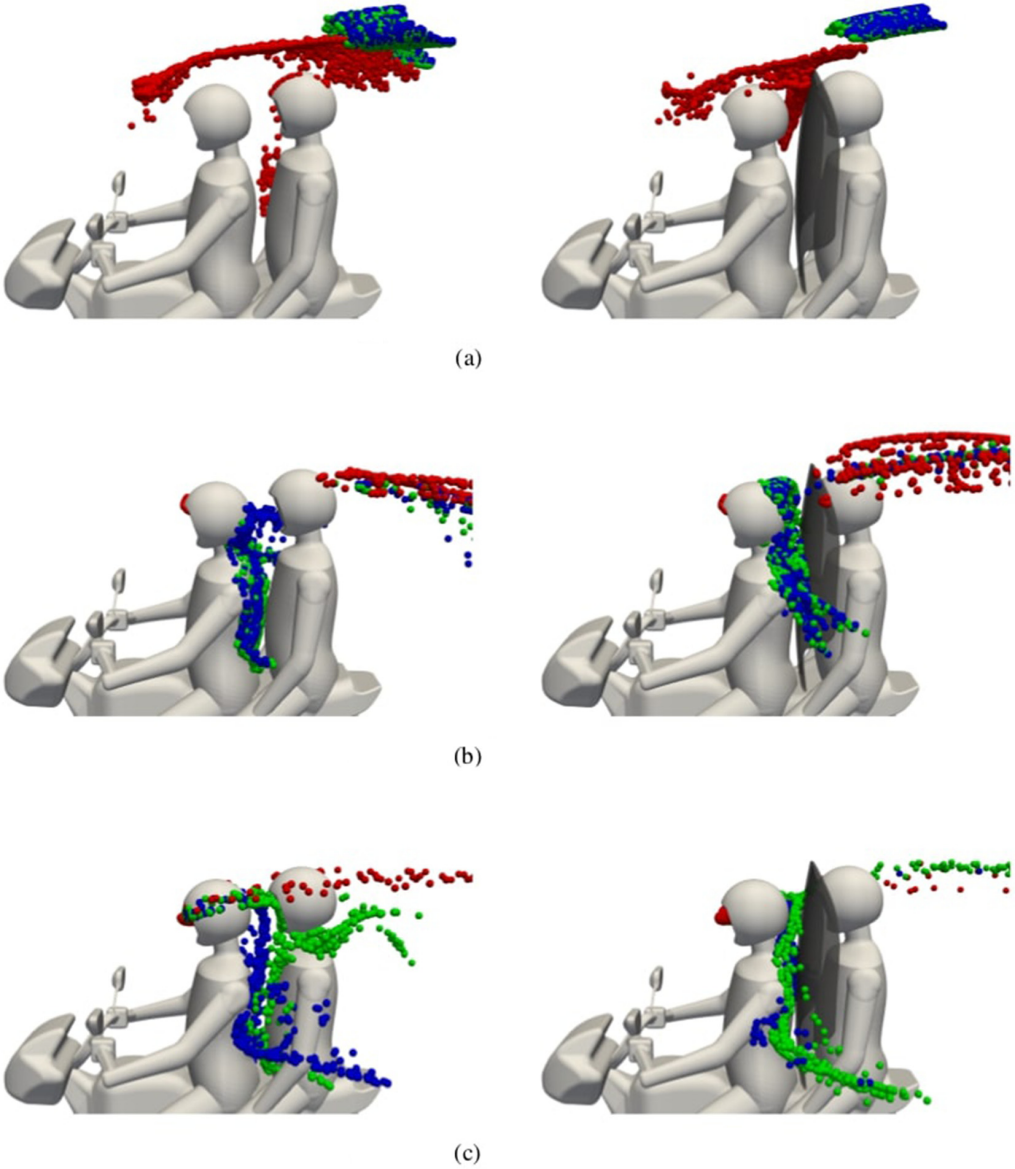
Particle distribution snapshots in time without (left plots) and with (right plots) shield. Particles are colored by diameter $D_{p}$ according to blue ( 1), green ( 10), and red ( 50 μm). Motorbike travel speed and snapshot time: (a) , (b) $U_{\infty }=5\;m/s,\;t=0.5\;s$, and (c) $U_{\infty }=15\;m/s,\;t=0.2\;s$.

Particle distributions for each simulation are visualized in Fig. 7. Each snapshot was chosen at a specific time in the simulation where a large number of particles are located in close proximity to the passenger. Particles have been scaled up in size to aid visibility and colored by their diameter according to blue ( 1), green ( 10), and red ( 50 μm).

At the lowest speed of $U_{\infty }=1\;m/s$, smaller particles ( $D_{p}\leq 10\;\mu m$) are ejected far enough away from the driver and are unable to settle under gravity back toward the riders. Instead, they are carried over the rider's heads and pose no risk. This can be explained by the prominent jet from the driver's mouth, as seen in Fig. 5. In contrast, large particles ( $D_{p}=50\;\mu m$) are carried back toward the passenger, fall under gravity, and settle between the riders. The addition of a shield appears to have blocked some of those particles from hitting the front of the passenger and has created a desirable flow field around the passenger's head which carries particles further away.

When the inlet speed is increased to 5 and 15 m/s, large particles ( $D_{p}=50\;\mu m$) either travel straight back into the rider's face or are carried away from the passenger. However, a significant problem exists for the smaller particles at these speeds. A recirculation region is present behind the driver which carries small particles into the gap between riders. There are two risks associated with this: (i) particles will deposit onto the passenger and (ii) particles will be inhaled by the passenger. This isn't a problem with the largest particles because they are too big to follow smaller particles into the recirculation region. When a shield is added, particles are still able to travel into this gap, but are pushed further away from the passenger's head.

Table III presents the deposition of particles onto different surfaces, as a percentage of the total number injected into the domain. A total of 1004 particles are injected into the domain in the time window 0≤t (s)≤0.3. For most cases, particles are washed away from the geometry and toward the outlet after only 5 s. However, in some cases, a region of recirculation is present behind the geometry, causing particles to wash back toward the passenger. For these cases, simulations are run until all particles have either settled out of suspension or exited the domain through the outlet patch.

**TABLE III.** Deposition of particles from each case, as a percentage of the total number injected into the domain.

	$U_{\infty }$ (m/s)	$D_{p}(\mu \;m)$	Surface deposition (%)			
			Driver	Shield	Passenger	
					Helmet	Body
No shield	1	1	0	⋯	0	0
		10	0	⋯	0	0
		50	0	⋯	2.2	2.9
	5	1	0.2	⋯	0	0
		10	7.2	⋯	0	0.1
		50	47.1	⋯	0	0
	15	1	0.1	⋯	0	0.1
		10	0	⋯	29.4	0.3
		50	72.2	⋯	0	0
Shield	1	1	0	0	0	0
		10	0	0	0	0
		50	34.4	1.4	5.3	2.1
	5	1	0	0	0	0
		10	1.0	0	0	0
		50	41.9	4.9	0	0
	15	1	0	0	0	0
		10	0	0	0	0
		50	88.3	0	0	0

General trends in the data for the case without a shield are discussed first. There is very little deposition of $D_{p}=1\;\mu m$ particles—at most only 0.2% of the total injected into the system is deposited, which occurs for speeds of $U_{\infty }=5$ and 15 m/s. This is surprising because Fig. 7 shows how small particles reside in the gap between riders. An explanation is offered here—smaller particles behave more like flow tracers and are unable to penetrate the boundary layer and deposit onto surfaces. However, although $D_{p}=1\;\mu m$ particles are not depositing onto the riders, it is still undesirable for them to enter the gap between the riders because the passenger may inhale particles.

For the intermediate size 10 μm, there is some deposition for speeds of 5 and 15 m/s. At 5 m/s, 7.2% is deposited onto the driver, compared to no deposition at 15 m/s. However, at 15 m/s, a significant portion is deposited onto the passenger's helmet (29.4%). From Fig. 7, it is clear that an intermediate size of 10 μm travels toward and around the helmet and neck of the passenger, which leads to a significant amount of deposition. Once a shield is added, these particles take a different route—first traveling down the shield, then out toward the side, and away from the passenger.

More deposition occurs for larger particles, especially onto the driver. Larger particles have greater inertia; therefore, once the rider coughs, particles are swept straight back onto their face and helmet. Additionally, deposition of $D_{p}=50\;\mu m$ particles onto the rider increases as inlet speed increases. An inlet speed of 15 m/s is greater than the coughing speed of 10 m/s, so therefore particles will not travel far enough away from the rider, and instead will wash straight back into the rider's helmet. For the largest particles at an inlet speed of 1 m/s, some deposition onto the passenger's helmet and body occurs with and without a shield. This occurs as particles travel over the shield and settle under gravity into the low velocity region immediately above the passenger's helmet.

### C. Infection risk of sub-micron aerosols

It has been shown that deposition onto the passenger is negligible for the smallest diameter particle tested here ( $D_{p}=1\;\mu m$). However, Fig. 7 highlights the possible risk of the smallest particles in the system, which follow flow streamlines and can become trapped in the gap between the two riders. A shield can be seen to push particles back toward the driver, but particles still travel into the gap. It is not sufficient to only consider those particles which are deposited onto the passenger because the passenger can inhale aerosols into their mouth from a small volume in front of their face. To account for those particles which may not be deposited, but may still be inhaled, Liu *et al.*^11^ defined an exposure region in front of the susceptible individual by a sphere with volume $0.002\;m^{3}$.

**FIG. 8.**
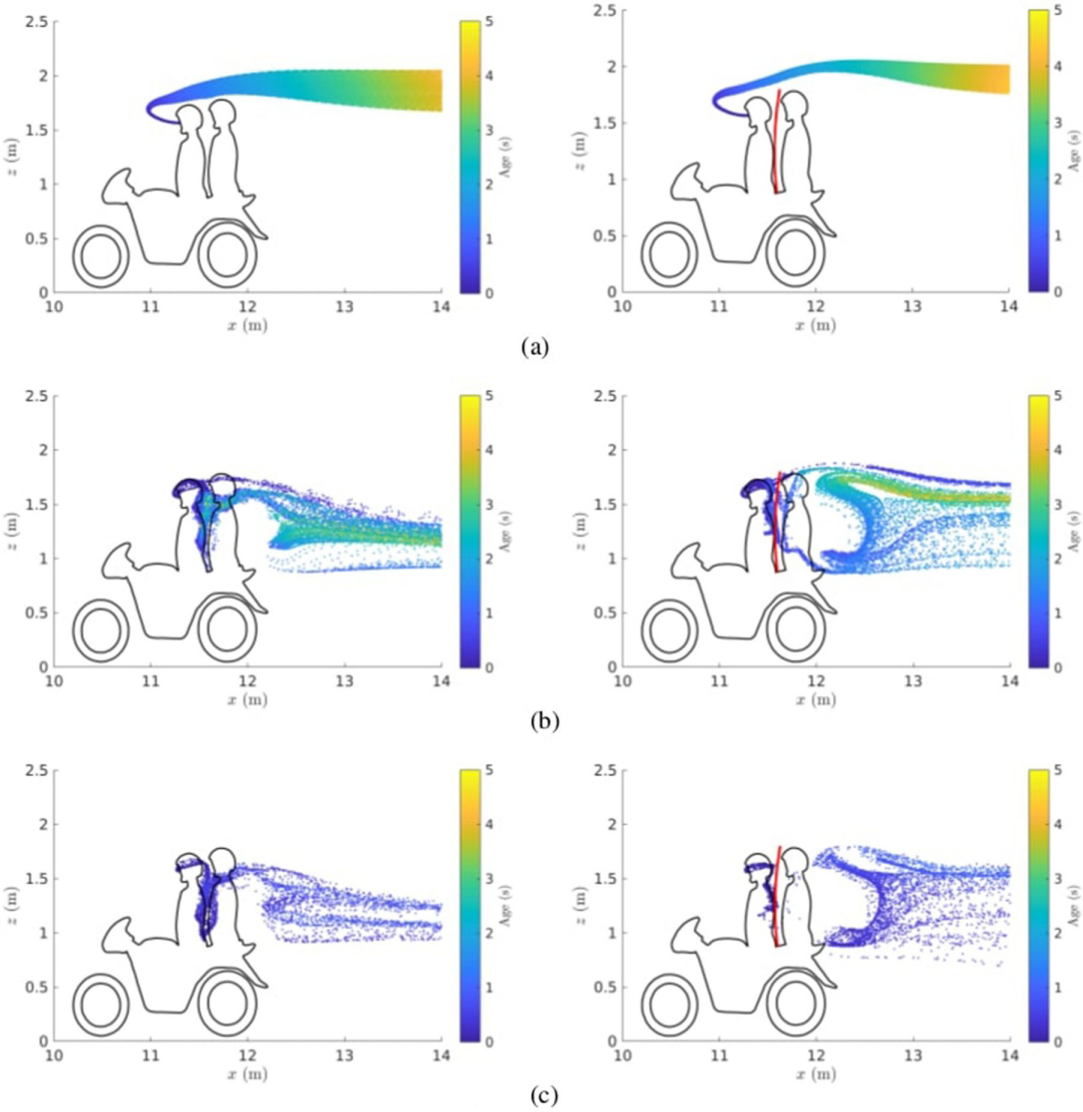
Distribution of particles without (left plots) and with shield (right plots) for a time window 0<t (s)<5, along a slice in the *x*-*z* plane centered around *y *=* *0, with thickness 0.47 m. Motorbike travel speed: (a) $U_{\infty }=1$, (b) $U_{\infty }=5,$ and (c) $U_{\infty }=15\;m/s$.

To visualize the paths taken by 1 μm particles which travel in close proximity to the passenger, a slice of thickness 0.47 m is taken in the *x*-*z* plane, centered around *y *=* *0. This is equivalent to the diameter of a sphere with volume $0.002\;m^{3}$, defined in Liu *et al.*^11^ as the exposure region. Figure 8 displays particle positions for every time step in the window 0<t (s)<5, colored by particle age. At the lowest speed 1 m/s, particles are carried over the passenger's head. For the case with a shield, the stream of particles is pushed further away from the passenger. However, for the intermediate and high speed inlet cases of 5 and 15 m/s, particles build up in between the driver and passenger. For the case where no shield is used at inlet speed 5 m/s, some particles in between the riders are aged t≈5 s.

**FIG. 9.**
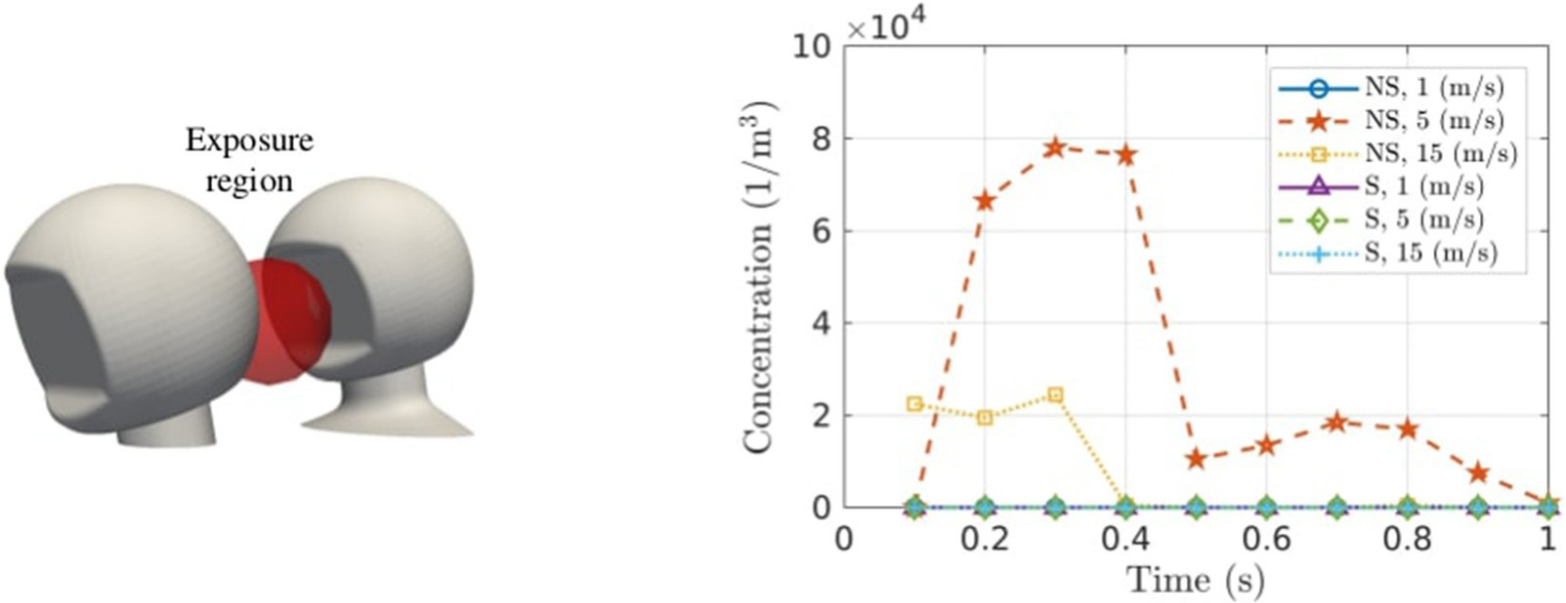
Left: exposure region defined by a sphere of volume $0.002\;m^{3}$ in front of the passenger's face. Right: concentration of particles in the exposure region as a function of time.

In order to count the number of particles traveling through the exposure region defined in Liu *et al.,*^11^ solution files are written out at a time step of 0.1 s. Particle coordinates and particle age are available at each time step and processed in MATLAB. Figure 9 displays the exposure region in front of the passenger's face and plots the concentration of particles in the sphere as a function of time. Only 1 μm particles are considered. For cases with a shield, the exposure region is split approximately in half, and only those particles which pass into the half closest to the passenger are registered. No particles are registered in the exposure region when a shield is used. However, for the cases when no shield is used, particles are registered at speeds of 5 and 15 m/s. Two peaks are present in the profile for 5 m/s. The first peak at t≈0.3 s corresponds to an influx of particles straight from the driver's mouth. This is followed by a sharp decrease in concentration when particles travel downwards in between the riders. A second peak at t≈0.7 s registers as the particles flow back up toward the passenger's mouth.

It is clear from Figs. 8 and 9 that aerosols are capable of following the flow streamlines into recirculation regions, at which point they might reside in that region for a considerable amount of time. For the case of a ride-sharing motorbike taxi, the recirculation region behind the driver pulls aerosols in, which causes a risk to the passenger of transmission, via virus inhalation. A shield does not work as a barrier to aerosols, but instead alters the flow field in between the riders, and pushes aerosols away from the passenger.

## IV. CONCLUSIONS AND RECOMMENDATIONS

In this paper, a CFD-based investigation has been undertaken to model particle transport from an infected driver to a passenger in a motorbike taxi setting. A single cough is modeled from the driver, which is transported back toward the passenger by the surrounding air flow. The motorbike is assumed to be traveling at three different speeds: 1,5,15 m/s. A complex turbulent flow is generated by the motorbike and riders, which heavily influences the transport of particles. Three particle diameters are tested to represent the full range of behavior—from small aerosols to large ballistic projectiles.

Two modes of transmission are investigated here: (i) deposition onto the passenger and (ii) inhalation of particles directly in front of the passenger. Large particles $D_{p}=50\;\mu m$ pose a risk to the passenger at lower speeds ( $U_{\infty }\approx 1\;m/s$) because the jet generated by a cough dominates the background flow, carrying particles over the driver and onto the passenger. When the motorbike is traveling at greater speeds ( $U_{\infty }≳5\;m/s$), large particles are deposited directly back onto the driver. However, aerosols pose a different risk to the passenger because very little deposition occurs. Instead, aerosols are shown to travel in between the driver and passenger due to the recirculation region present between the riders. A susceptible region is defined in front of the passenger, and the concentration of particles registered in this region is significant for the cases of a motorbike speeds $U_{\infty }=5$ and 15 m/s. Visualizations have shown that particles are pulled into this region and may reside for some time until they are finally washed out.

A shield is added between the driver and passenger to reduce the risk exposure for the passenger. The shield works in two ways: (i) physically blocking large particles from depositing onto the passenger and (ii) creating a favorable flow between the riders which flushes aerosols away from the passenger. Given the immense economic, political, and social pressure for the motorbike taxis to be continued during the COVID-19 and similar such epidemics and pandemics, the findings of this study suggest that a shield be fitted between the driver and the passenger. This reduces the risk associated from deposition of large particles, and inhalation of aerosols. Although at low speeds generally encountered in urban traffic, the driving safety should not be compromised, studying the stability of the motorbikes with the shields is suggested as a future avenue of research.

## Figures and Tables

**FIG. 10.**
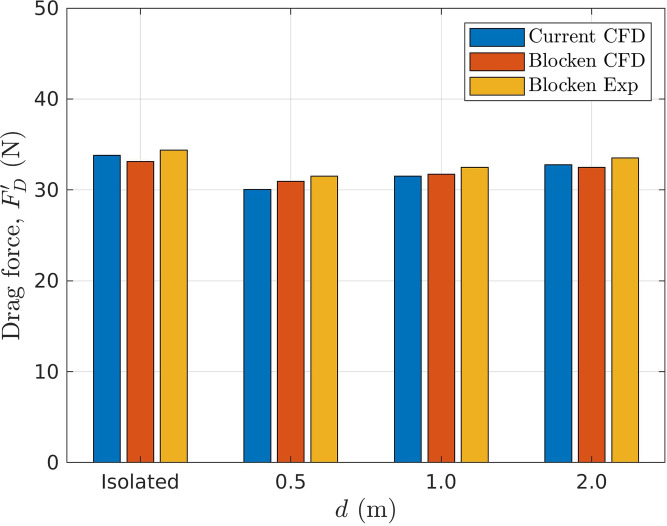
Drag force of a cyclist trailed by a motorcycle as a function of separation distance *d*. Experimental and CFD data from Blocken *et al.*^37^

## Data Availability

The data that support the findings of this study are available from the corresponding author upon reasonable request.

## APPENDIX: VALIDATION

The combined experimental and numerical study of Ref. 37 has been chosen to validate the current CFD method for a number of reasons. First, both experimental and CFD data were made available in their study. Second, their flow field is of similar nature to the problem of a motorbike taxi, i.e., external aerodynamic flow driven by separation.^37^ The drag reduction of a cyclist followed by one, two, and three motorbikes is considered. The bulk flow speed was 15 m/s, which matches the top speed in the current investigation. The separation distance *d* between the cyclist's back tire and the motorbike's front tire was varied. Here, validation against Blocken *et al.*^37^ is carried out for only one case—a cyclist followed by one motorbike.

The computational domain matches that of Blocken *et al.*^37^—the domain bounding box extends 10.2 m upstream of the cyclist's front wheel, ± 10.6 m either side in the normal direction *y*, and is 12.4 m in height. Table IV presents details of the meshes generated and the predicted drag force for a cyclist followed by a single motorbike at d=1.0 m. The computational time for each simulation to complete 5000 iterations has been denoted *T*_5000_, and it can be seen that simulation time increases approximately linearly with respect to the total number of cells. Drag predicted by each mesh is compared to the results of Blocken *et al.*,^37^ where $\langle F_{D}^{\prime }\rangle =31.7\;N$ was reported in their *k*-*ε* RANS model, and $\langle F_{D}^{\prime }\rangle =32.5\;N$ was reported in their wind tunnel experiment. An increase in drag from CFD to experimental results was accredited to additional drag from supports mounting the cyclist model in the wind tunnel. A percentage difference has been calculated in relation to the RANS results of Blocken. Close agreement between the current study and^37^ is observed for meshes M4, M5, and M6. A linear decrease in $\left\langle F_{D}^{\prime }\right\rangle$ is observed until approximately 31 million cells, after which it becomes constant, even with further refinement. It can be seen that a minimum of 31 × 10^6^ cells are required to predict drag within 1% of the value reported in Blocken *et al.*^37^

**TABLE IV.** Details of the 6 meshes used to determine mesh sensitivity for the case of a cyclist followed by a single motorbike at separation d=1.0 m. *T*_5000_: clock time in minutes to reach 5000 iterations. Percentage difference is calculated in relation to the RANS results of Blocken *et al.*,^37^ who reported $\langle F_{D}^{\prime }\rangle =31.7\;N$.

Mesh	Cells (10^6^)	*T*_5000_ (mins)	$\left\langle F_{D}^{\prime }\right\rangle$ (N)	% Diff.
M1	6.8	165	34.6	+9.1
M2	14.4	206	33.6	+6.0
M3	22.0	563	32.8	+3.5
M4	31.7	850	31.5	−0.6
M5	39.5	1035	31.4	−0.9
M6	47.8	1312	31.4	−0.9

To finish validation of the current numerical method, the separation distance is varied between d=0.5,1.0,2.0 m. A suitable mesh for each case is generated automatically from the method developed previously. This is possible because the gap between cyclist and motorbike is always refined to the same level, regardless of *d*. A summary of the drag force experienced by the cyclist for a trailing motorbike with respect to *d* is presented in Fig. 10. Reasonable agreement is found for all separation distances tested here. Drag predicted in the current simulations for an isolated cyclist sits somewhere between the CFD and experiments of Blocken *et al.*^37^ $F_{D}^{\prime }$ is slightly under-predicted at distance d=0.5 m in the current CFD, but as *d* is increased, $F_{D}^{\prime }$ reported in the current results increases beyond the drag predicted by the CFD results of Blocken and approaches the experimental results. The reasons for this are unclear, but this suggests that the current mesh and turbulence model have more accurately predicted the flow between the cyclist and motorbike.
